# Supplementary material for: Complexome profiling of the Chlamydomonas psb28 mutant reveals TEF5 as an early PSII assembly factor
Source: Plant Cell. 2025 Mar 17;37(6):koaf055. doi: 10.1093/plcell/koaf055 (PMC12164590; doi:10.1093/plcell/koaf055)
Supplement: koaf055_Supplementary_Data [file koaf055_supplementary_data.zip › psb28_interactive.zip!/plots/intensity/qualityPlots/peptides/CGL3_Cre05.g245102.t1.1.html]

### Cre05.g245102.t1.1

Trivial name: CGL3  
SeqCoverage [median]: 27.30  
Mass: 19.02 kDa  
  
 

| Sequence | MissedCleavage | Mass [Da] | wtIntensity | muIntensity | Score |
| --- | --- | --- | --- | --- | --- |
| FVGPALGLYK | 0 | 1063.61 | 0.00 | 1360.53 | 62.66 |
| GPAPGPAASRPPSAGMPGAK | 1 | 1772.90 | 0.00 | 1171.63 | 40.52 |
| LAGEFAPPPV | 0 | 996.53 | 728.63 | 0.00 | 135.87 |
| LTELYLGR | 0 | 963.54 | 1232.73 | 2714.85 | 134.66 |
|  |  |  | 1961.4 | 5247.0 |  |
